# Ciliated conical epithelial cell protrusions point towards a diagnosis of primary ciliary dyskinesia

**DOI:** 10.1186/s12931-018-0782-3

**Published:** 2018-06-25

**Authors:** Chris O’Callaghan, Andrew Rutman, Gwyneth Williams, Neeta Kulkarni, Joseph Hayes, Robert A. Hirst

**Affiliations:** 1 Respiratory, Critical Care and Anaesthesia, UCL Great Ormond Street Institute of Child Health & Great Ormond Street Children’s Hospital & NIHR Great Ormond Street Hospital Biomedical Research Centre, 30 Guilford Street, London, WC1N 1EH UK; 2 Department of Infection, Centre for PCD Diagnosis and Research, Immunity and Inflammation, RKCSB, University of Leicester, Leicester, LE2 7LX UK

**Keywords:** Respiratory cilia, PCD, Asthma, Severe asthma, Cystic fibrosis, Diagnostic testing, Primary ciliary dyskinesia

## Abstract

**Background:**

Primary ciliary dyskinesia can result from a number of different ciliary defects that adversely affect ciliary function resulting markedly reduced or absent mucociliary clearance. Improvement in diagnostic testing is an area of current research. During diagnostic evaluation of PCD we observed ciliated conical protrusions from part of the apical surface of ciliated cells in those diagnosed with PCD. The aim of this study was to investigate if this abnormality was specific to PCD.

**Methods:**

Epithelial edges from 67 consecutively diagnosed PCD patients, 67 patients consecutively referred for PCD diagnostic testing in whom PCD was excluded, 22 with asthma and 18 with Cystic Fibrosis (CF) were studied retrospectively in a blinded manner using light microscopy.

**Results:**

Forty six out of 67 patients with PCD had ciliated conical epithelial protrusions, whereas none were seen in patients where PCD was excluded, or in patients with asthma or CF. The sensitivity, specificity, positive predictive value and negative predictive value for the presence of the ciliated conical protrusions to predict a diagnosis of PCD were 76.5, 100, 100 and 77% respectively.

**Conclusions:**

Characteristic ciliated conical protrusions from ciliated epithelial cells maybe a useful pointer to the diagnosis of PCD. However, their absence does not exclude the diagnosis of PCD.

**Electronic supplementary material:**

The online version of this article (10.1186/s12931-018-0782-3) contains supplementary material, which is available to authorized users.

## Background

Primary Ciliary Dyskinesia (PCD) is a rare autosomal recessive disease affecting motile cilia throughout the body. The prevalence has been reported at 1 in 2200 in a highly consanguineous British Asian population [1] with estimates in Europe ranging from 1 in 10,000 to 1 in 40,000 [2, 3]. Over 35 genes have been discovered that cause PCD [4] accounting for up to 65% of PCD patients [5]. PCD has a profound effect on mucociliary transport with those affected suffering from a daily wet sounding cough and nasal symptoms from the newborn period. Significant numbers develop glue ear with associated hearing problems and approximately 45% have *situs inversus*. Bronchiectasis may develop in early childhood and severe lung disease is not uncommon in older patients [4]. Many patients with PCD remain undiagnosed or are diagnosed late after years of chronic respiratory symptoms.

There is no gold standard diagnostic test for PCD [5]. Diagnostic testing of those with a suggestive clinical history may include nasal nitric oxide (nNO) measurement [5], high-speed video microscopy to assess ciliary beat pattern and measure ciliary beat frequency [6] and electron microscopy [5]. Diagnostic testing can be difficult due to secondary ciliary dyskinesia although this problem may be markedly reduced by testing at least 4 weeks after a viral upper respiratory tract infection [7, 8]. Culture of nasal epithelial samples to an air-fluid interface can be helpful in some cases as it significantly reduces secondary ciliary dyskinesia seen in the original sample but maintains, and in many cases exaggerates, the abnormal beat pattern seen in original samples [9, 10]. The place of other tests including gene testing and immunofluorescence [11–13] are being evaluated to determine if they can help improve diagnostic certainty.

During diagnostic evaluation of patients suspected of PCD, we observed ciliated conical protrusions from the surface of ciliated epithelial cells in nasal epithelial samples from many patients diagnosed with PCD by light and electron microscopy. We therefore formally studied the presence of ciliated conical protrusions from the epithelium of patients with PCD to test the hypothesis that their presence was predictive of the diagnosis. To determine if the ciliated conical protrusions were specific to PCD patients we also studied respiratory epithelial samples from patients referred for diagnostic testing for PCD where the diagnosis was excluded, patients with mild, moderate and severe asthma and patients with cystic fibrosis.

## Methods

### Subjects

Nasal brush biopsies from the following groups were retrospectively analysed: 67 consecutively diagnosed patients with PCD over a 2 year period by the National PCD Diagnostic Clinic, Leicester, UK: 67 consecutive patients referred for diagnostic testing for PCD where PCD was excluded; 22 patients with well-characterised asthma (11 severe, 6 moderate, 5 mild) and 18 patients with cystic fibrosis (CF).

### Nasal brush biopsies

Ciliated epithelium was obtained by a single gentle brushing of the inside of the nose as previously described [14]. Brushings were only performed if patients declared themselves free from an upper respiratory tract infection for at least 1 month. Cells obtained were suspended in 2 ml of HEPES (20 mM) buffered medium 199 containing penicillin (50 μg/ml), streptomycin (50 μg/ml) and Fungizone (1 μg/ml). For each patient, the sample was divided in to three to allow, initial ciliary functional analysis, fixation for transmission electron microscopy (TEM) and cell culture.

### Epithelial assessment

All samples were studied using the PCD diagnostic center’s protocol. Ciliated epithelial edges were observed using a × 100 oil immersion lens and recorded using a high-speed video camera allowing ciliary beat pattern and beat frequency to be determined, as previously described [15]. Images from videos were studied to determine the presence or absence of ciliated conical protrusions. If a high degree of secondary damage was seen the samples were cultured to an air fluid interface to allow reassessment of ciliary function after differentiation.

**Fig. 1:**
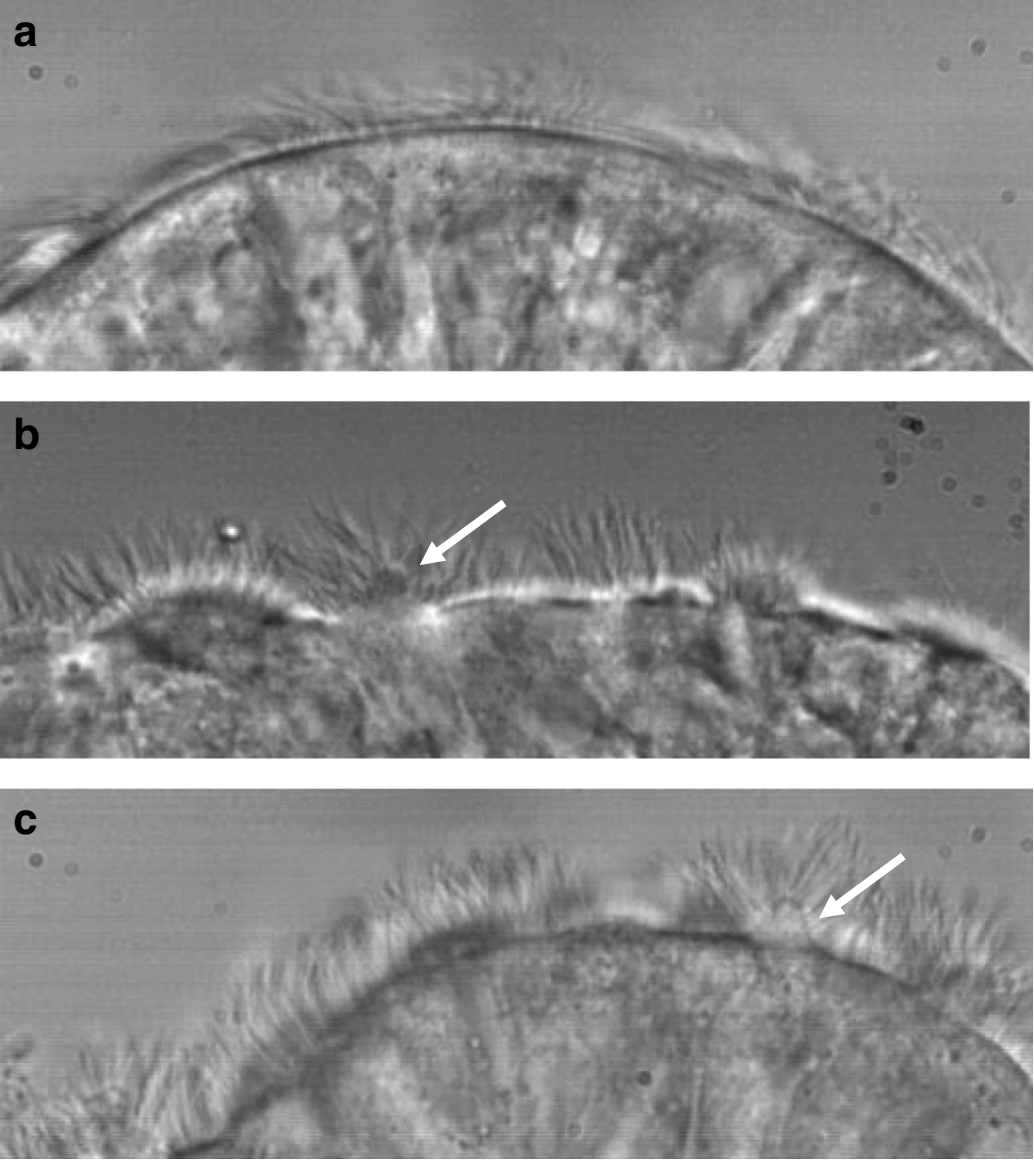
Single frame capture from a high-speed video showing ciliated epithelial edges observed using a 100× oil immersion lens. **a** Healthy control showing an intact ciliated epithelium. **b** and **c** Ciliated epithelium from a PCD sample showing characteristic conical ciliated projection (arrow)

**Fig. 2:**
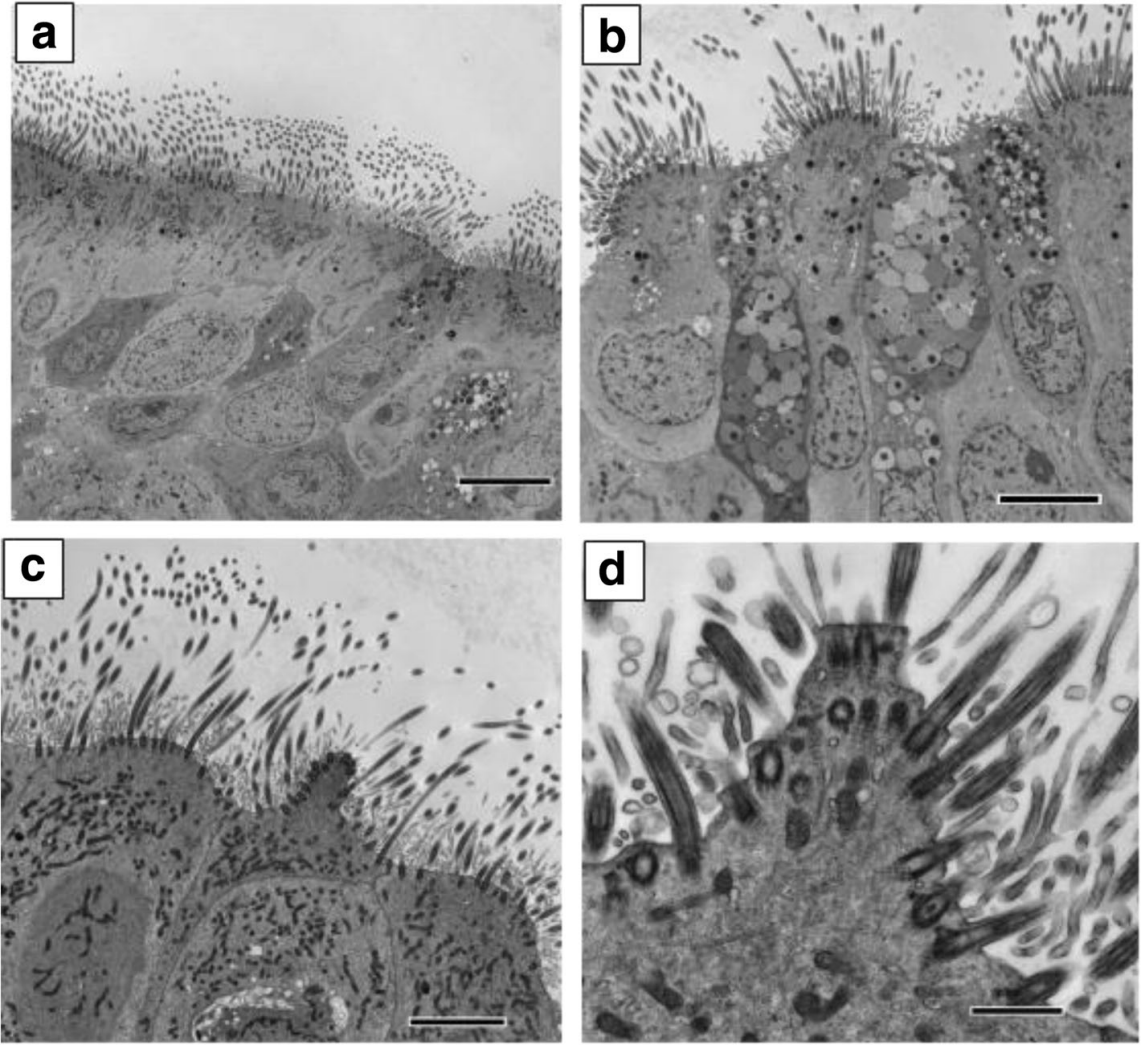
Transmission electron microscopy cross sections of ciliated respiratory epithelium showing; **a** Healthy well ciliated epithelium from a healthy control (bar = 8 μm). No protrusions were seen in the TEM images obtained from CF and Asthma patients. **b** Ciliated respiratory epithelium from a patient with bronchiectasis who had the diagnosis of PCD excluded. Ciliated cells are seen to project from the epithelium. Note the projection involves the whole surface of the cell and does not have a conical shape (bar = 6 μm). **c** Ciliated epithelium from a patient with PCD showing a characteristic ciliated conical protrusion (arrow) (bar = 4 μm). **d** High power of a ciliated conical protrusion (bar = 800 nm)

To be classified as a ciliated conical protrusion the following criteria were met: a) protrusions were conical in shape and ciliated, b) they project from part of the apical surface of a ciliated cell, c) the cell that has a conical protrusion is in normal alignment with adjacent cells with no evidence of disruption of tight junctions between adjacent cells on electron microscopy. They were differentiated from minor and major cell protrusions where the apical surface of the entire cell was seen to protrude from adjacent cells indicating disruption of intra cellular junctions. Where possible 7–10 ciliated epithelial edges of approximately 100 μm in length were studied for each patient. The appearances of ciliated conical protrusions can be seen on light microscopy in Fig. 1 and on electron microscopy in Fig. 2.

### Transmission electron microscopy (TEM)

The epithelium from fresh brush biopsies and cultured cells were fixed in glutaraldehyde (4%) and processed for TEM analysis as previously described [16].

### Ciliated cell culture

Re-growth of ciliated epithelium at an air-fluid interface from the initial brush biopsy was performed in 38 patients with PCD, in 11 of the patients referred for diagnostic testing for PCD in whom the diagnosis was excluded, in 10 patients with asthma and in 6 patients with cystic fibrosis. After 4 to 6 weeks the cultures were brushed to provide ciliated epithelial edges that were evaluated using the methods described to assess the initial brush biopsy. The method used for air liquid interface cultures has previously been described [10].

### Diagnostic testing for PCD

The diagnosis of PCD was based on a characteristic clinical picture of a life long ‘wet sounding’ cough and nasal symptoms with supportive diagnostic tests. PCD patients in this study were diagnosed on the minimum criteria of a) a characteristic clinical history, b) a beat pattern abnormality characteristic of PCD observed, using high speed video microscopy, and c) an ultrastructural defect consistent with PCD on transmission electron microscopy [6, 17, 18]. The diagnostic phenotypes are listed in Table 1.

**Table 1:** Ciliated conical protrusions observed using light microscopy and a × 100 lens before and after ciliated cell culture (− Cultured) at an air liquid interface. * Radial spoke defect in this paper is defined as absent inner dynein arms with microtubular disorganization

Phenotype	Number of subjects	Number of subjects with ciliated protrusions	Ciliated edges observed per subject Mean (SD)	Protrusions per ciliated edge Mean (SD)	Protrusions per subject Mean (SD)
All PCD Diagnosed	67	46	6.5 (1.8)	0.79 (0.6)	7.6 (7)
- Cultured	38	9	3.9 (2.4)	0.09 (0.3)	1.0 (3.1)
Radial spoke defect *	13	10	11 (1.9)	0.76 (0.6)	8.8 (7.3)
- Cultured	7	1	9.1 (2.2)	0.01 (0)	0.2 (0.4)
No inner dynein arms	13	6	2.3 (1.9)	0.31 (0.5)	3.3 (5)
- Cultured	6	1	0.2 (3.1)	0.03 (0.1)	0.2 (0.5)
No outer dynein arms	12	8	3.5 (1.4)	0.66 (0.7)	6.6 (7)
- Cultured	6	0	7.2 (1.5)	0 (0)	0.0 (0)
No dynein arms	16	11	9.5 (1.0)	0.74 (0.7)	7.2 (7.3)
- Cultured	13	3	0.9 (1.9)	0.09 (0.2)	0.7 (1.6)
Transposition	13	11	6.4 (2.0)	1.11 (0.5)	12.2 (5.7)
- Cultured	8	4	2.3 (2.6)	0.41 (0.6)	3.7 (6.3)
Referred: not PCD	68	0	7.8 (2.0)	0 (0)	0.0 (0)
- Cultured	11	0	7.2 (1.1)	0 (0)	0.0 (0)
Cystic Fibrosis	18	0	7.6 (1.4)	0 (0)	0.0 (0)
- Cultured	6	0	6.6 (1.8)	0 (0)	0.0 (0)
Asthma	24	0	8.9 (1.0)	0 (0)	0.0 (0)
- Cultured	10	0	7.1 (1.1)	0 (0)	0.0 (0)

### Statistics

Sensitivity, specificity, positive predictive values (PPV) and negative predictive values (NPP) were calculated for the presence of ciliated conical protrusions from epithelial cells for initial brush biopsies and after ciliated cell culture.

## Results and discusssion

Results are shown in detail in Table 1. The mean and standard deviation of the width and height of the ciliated conical protrusions were 3.4 ± 0.51 μm (max 4.3; min 2.1) and 3.9 ± 0.52 μm (max 5.1; min 2.8) respectively. A high speed video recording of the ciliated conical protrusions are shown in Additional file 1.

Ciliated conical protrusions were observed in the ciliated epithelium in 73% of patients diagnosed with PCD on the initial brush biopsy samples. Examination of the ciliated epithelium from these patients, after regrowth at an air liquid interface, showed a marked reduction in ciliated conical protrusions (73 to 15%).

No ciliated conical protrusions were seen in the 67 patients referred for diagnostic testing for PCD where the diagnosis was excluded, or in patients with CF or mild, moderate or severe asthma in the initial samples or following culture of ciliated epithelium at an air liquid interface.

The sensitivity, specificity, PPV and NPV for the presence of ciliated conical protrusions to predict a diagnosis of PCD were 76.5, 100, 100, and 77% respectively (for calculation see Additional file 2).

Typical images of the ciliary conical like protrusions on light microscopy are shown in Fig. 1 and on electron microscopy are shown in Fig. 2.

This study reports a novel observation that characteristic ciliated conical protrusions from the surface of ciliated cells are commonly seen in nasal epithelial samples from patients with PCD and are seen across different PCD phenotypes both on light and transmission electron microscopy. They were seen in both very healthy well ciliated strips of epithelium from patients with PCD and in areas where secondary epithelial damage was present. The number seen in patient samples was not associated with increasing patient age. We found the presence of ciliated conical protrusions had a specificity for PCD of 100%. Sensitivity however, was lower at 76.5%. Although fewer ciliate conical protrusions were seen following ciliated cell culture, their presence in cultures suggests they are not due to solely to underlying infection or external inflammatory stimuli. As ciliated cells lining the respiratory epithelium survive for many months, and ciliated cell cultures were studied after just 4 weeks in culture it is possible that the number of protrusions in cell cultures may have increased over time. It is also possible that respiratory infection or inflammation in the respiratory tract of PCD patients may have contributed to increased numbers of ciliated conical projections seen in epithelial samples taken directly from patients. In contrast to protrusion of cells from the epithelial surface (7), that is commonly seen in the presence of secondary epithelial damage [8], conical ciliated protrusions involve only a proportion of the surface of a ciliated cell and did not appear to be associated with breakdown of intracellular junctions.

The absence of ciliated conical protrusions from ciliated cells in patients with mild, moderate and severe asthma and CF, as well as in the epithelium of patients with chronic respiratory symptoms in whom the diagnosis of PCD was ruled out suggests that this feature may be specific to PCD.

The ciliated conical protrusions were seen in all phenotypes of PCD studied and were not directly related to the presence of static cilia as in some of the PCD phenotypes studied cilia beat frequency was within the normal range. We could find only one study on cell membranes in patients with PCD, by Kantar and colleagues [19]. This showed increased membrane fluidity in neutrophils from patients with PCD. The authors speculated that alterations in the cytoskeleton may be responsible, however, it was not possible to perform similar measurements in ciliated cells and the mechanism responsible for the ciliated conical protrusions and increased membrane fluidity in neutrophils remains unclear. We believe that a combination of increased membrane fluidity in PCD epithelial cells coupled with disruption in the cellular dynein cytoskeleton may cause the features that we see, however this remains to be proven.

The results of this study will not change our diagnostic approach that includes measurement of nasal nitric oxide in those old enough who have patent nasal passages, assessment of ciliary beat pattern and beat frequency by high speed video microscopy and electron microscopy, supplemented by immunofluorescence testing, ciliated cell culture and genetic testing as required. However, as multiple epithelial edges are observed during our initial routine diagnostic assessment the presence of ciliated conical protrusions from can be recorded and may help to increase confidence in diagnosis.

## Conclusion

In summary, our data shows that ciliated conical protrusions from ciliated cells in the respiratory epithelium are common in PCD. Their absence in asthma and CF, as well as in the epithelium of patients with chronic respiratory symptoms in whom the diagnosis of PCD was excluded suggests that they point towards the diagnosis of PCD.


Additional file 1:**Video S1.** The first epithelial edge in the video is taken from a patient with PCD due to absent inner dynein arms and disorganised microtubules (Radial spoke defect). The video is in real time and shows all of the cilia are dyskinetic. The arrows point towards two conical ciliated epithelial projections. The second video is included to show a close up of a ciliated conical epithelial projection on the left side and to contrast with it shows a cell protruding from the epithelium on the right side. (MP4 61,535 kb)


## Additional files

Additional file 2: Sensitivity calculation. (DOCX 147 kb)

## References

[1] O'Callaghan C, Chetcuti P, Moya E (2010). High prevalence of primary ciliary dyskinesia in a British Asian population. Arch Dis Child.

[2] Azfelius BA, Stenram U (2006). Prevalence and genetics of immotile cilia syndrome and lefthandedness. Int J Develop Biol.

[3] Torgersen J (1947). Transposition of viscera – bronchiectasis and nasal polypsgenetical analysis and contribution to the problem of constitution. Acta Radiol.

[4] Noone PG, Leigh MW, Sannuti A, Minnix SL, Carson JL, Hazucha M (2004). Primary ciliary dyskinesia: diagnostic and phenotypic features. Am J Respir Crit Care Med.

[5] Lucas JS, Barbato A, Collins SA, Goutaki M, Behan L, Caudri D (2016). European Respiratory Society guidelines for the diagnosis of primary ciliary dyskinesia. Eur Respir J.

[6] Chilvers MA, Rutman A, O’Callaghan C (2003). Ciliary beat pattern is associated with specific ultrastructural defects in primary ciliary dyskinesia. J Allergy Clin Immunol.

[7] Thomas B, Rutman A, O’Callaghan C (2009). Disrupted ciliated epithelium shows slower ciliary beat frequency and increased dyskinesia. Eur Respir J.

[8] Chilvers MA, McKean M, Rutman A, Myint BS, Silverman M, O’Callaghan C (2001). The effects of corona virus on human nasal ciliated respiratory epithelium. Eur Respir J.

[9] Hirst RA, Rutman A, Williams G, O’Callaghan C (2010). Ciliated air-liquid cultures as an aid to diagnostic testing of primary ciliary dyskinesia. Chest.

[10] Hirst RA, Jackson CL, Coles JL, Williams G, Rutman A, Goggin PM (2014). Culture of primary ciliary dyskinesia epithelial cells at air-liquid interface can alter ciliary phenotype but remains a robust and informative diagnostic aid. PLoS One.

[11] Shoemark A, Frost E, Dixon M, Ollosson S, Kilpin K, Patel M (2017). Accuracy of immunofluorescence in the diagnosis of primary ciliary dyskinesia. Am J Respir Crit Care Med.

[12] Olbrich H, Cremers C, Loges N, Werner C, Nielsen KG, Marthing JK (2015). Loss of function of GAS8 mutations cause primary ciliary dyskinesia and disrupt the nexin dynein regulatory complex. Am J Hum Genet.

[13] Adrien Fommer HR, Loges NT, Edelbusch C, Jahnke C, Raidt J (2015). Immunofluorescence analysis and diagnosis of primary ciliary dyskinesia with radial spoke defects. Am J Respir Cell Mol Biol.

[14] Chilvers M, O’Callaghan C (2000). Analysis of ciliary beat pattern and beat frequency using digital high-speed imaging: comparison with the photomultiplier and photodiode methods. Thorax.

[15] Chilvers M, Rutman A, O’Callaghan C (2003). Functional analysis of cilia and ciliated epithelial ultrastructure in healthy children and young adults. Thorax.

[16] Chilvers MA, Rutman A, O'Callaghan C (2003). Functional analysis of cilia and ciliated epithelial ultrastructure in healthy children and young adults. Thorax.

[17] Stannard W, Chilvers M, Rutman A, Williams CD, O’Callaghan C (2010). Diagnostic testing of patients suspected of primary ciliary dyskinesia. Am J Respir Crit Care Med.

[18] Stannard W, Rutman A, Wallis C, O’Callaghan C (2004). Central microtubular agenesis causing primary ciliary dyskinesia. Am J Respir Crit Care Med.

[19] Kantar A, Oggiano N, Giorgi PL, Fiorini R. Membrane fluidity of polymorphonuclear leukocytes from children with primary ciliary dyskinesia. Pediatr Res. 1993;34(6):725–8. 10.1203/00006450-199312000-00006 8108183

