# Supplementary figures and images for: Ciliated conical epithelial cell protrusions point towards a diagnosis of primary ciliary dyskinesia
Source: Respir Res. 2018 Jun 25;19:125. doi: 10.1186/s12931-018-0782-3 (PMC6019300; doi:10.1186/s12931-018-0782-3)

Sensitivity calculation


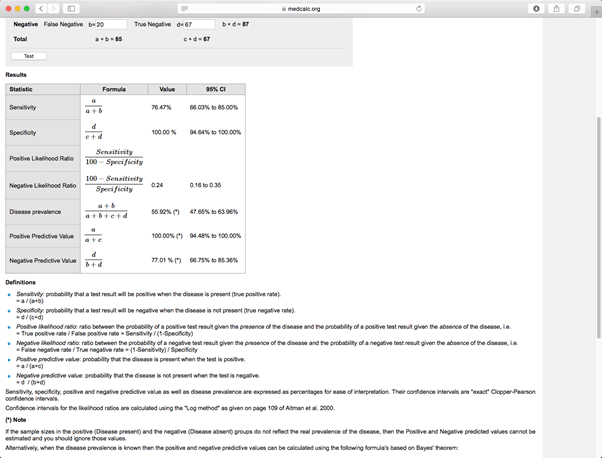

Supplement: Supplementary file 2 — Sensitivity calculation. (DOCX 147 kb) [file 12931_2018_782_MOESM2_ESM.docx]
